# Lumbar microdiscectomy and post-operative activity restrictions: a protocol for a single blinded randomised controlled trial

**DOI:** 10.1186/s12891-017-1681-3

**Published:** 2017-07-20

**Authors:** Chris D. Daly, Kai Zheong Lim, Jennifer Lewis, Kelly Saber, Mohammed Molla, Naor Bar-Zeev, Tony Goldschlager

**Affiliations:** 1 Department of Surgery, Monash University, Clayton, VIC Australia; 2 Department of Neurosurgery, Monash Medical Centre, Clayton, VIC Australia; 3 The Ritchie Centre, Hudson Institute of Medical Research, Monash University, Clayton, VIC Australia; 4 Department of Physiotherapy, Monash Medical Centre, Clayton, VIC Australia; 5 Centre for Global Vaccine Research, Institute of Infection & Global Health, University of Liverpool, Liverpool, UK; 6 Department of Neurosurgery, Cabrini Hospital, Malvern, VIC Australia

**Keywords:** Lumbar Discectomy, Spine, Restrictions, Post-Operative, Sitting

## Abstract

**Background:**

Lumbar microdiscectomy is the most commonly performed spinal surgery procedure, with over 300,000 cases performed annually in the United States alone. Traditionally, patients were advised to restrict post-operative activity as this was believed to reduce the risk of disc reherniation and progressive instability. However, this practice would often delay patients return to work. In contemporary practice many surgeons do not restrict patient post-operative activity due to the perception this practice is unnecessary. We describe a randomised controlled trial to assess the impact of activity restrictions on clinical outcome following lumbar discectomy.

**Methods/Design:**

The lumbar microdiscectomy and post-operative activity restriction trial is a multi-centre, randomised, controlled single blinded trial. Two hundred ten patients due to undergo single level lumbar microdiscectomy without a history of previous spine surgery, infection or fracture are randomised to be advised either restricted or unrestricted activity for a period of 30 days following lumbar microdiscectomy. Actual adherence with trial allocation will be monitored bioelectronically via a wearable device. Outcome assessment at follow up will occur at 1, 3, 6 and 12 months. The primary outcome will be a composite endpoint comprising changes in Visual Analogue Scale (Leg and Back), Oswestry Disability Index and the absence of intervertebral disc reherniation or secondary intervention.

**Discussion:**

This randomised controlled trial will directly compare post-operative protocols of activity restrictions and no restrictions following lumbar discectomy with adherence monitored bioelectronically.

**Trial Registration:**

Australian New Zealand Clinical Trials Registry: ACTRN12616001360404 (retrospectively registered 30/09/2016).

## Background

Lumbar microdiscectomy is the most commonly performed spinal surgical procedure [1]. Lumbar microdiscectomy is indicated for radicular pain unresponsive to conservative management (e.g. analgesia and physiotherapy), neurological deficit (e.g. weakness) or for cauda equina syndrome.

Lumbar microdiscectomy is minimally invasive, patients typically mobilize the same day and are discharged home the following day, making the operation suitable for day-procedure [2]. Traditionally following surgery, patients have been advised to restrict sitting, lifting or resuming other activities of everyday life, and are advised to either stand or lie for variable periods [3]. Sitting imposes greater intradiscal pressure than does standing [4] though evidence that increased pressure increases disc reherniation risk is lacking.

Such restrictions impact upon patients’ ability to return to work, travel or drive and basic comfort. It has been suggested that activity restrictions may also raise patient anxiety regarding reherniation risk. Moreover, neurosurgical practice regarding activity restriction varies, the dearth of evidence resulting in absence of clear clinical guidelines for surgeons, nurses, physiotherapists and occupational physicians. If no difference in outcomes are observed between groups in this randomised controlled trial future patients would be able to rapidly resume their normal activities, productivity, work and do so without fear or associated psychological morbidity. This would provide an evidence base to postoperative care and consensus amongst surgeons.

Two prospective studies published in the 1990s reported incidence of symptomatic recurrent disc protrusions and reoperation, and time to return to work in a cohort of patients whose movement was not restricted post lumbar microdiscectomy. Compared to rates in the literature among movement-restricted patients, adverse outcomes in this cohort were not considered higher [5, 6]. However, in the absence of a control group and randomisation, the evidence from such studies is relatively weak.

Bono et al. [7] recently published the first report of a randomised controlled trial investigating post-operative activity restrictions following lumbar discectomy. This trial compared post-operative protocols consisting of short (two weeks) and long (six weeks) periods of activity restriction following lumbar discectomy. The authors observed no significant difference in outcome as assessed by Visual Analog Scale (VAS) back or leg pain or Oswestry Disability Index (ODI). Disc reherniation rates differed between the groups observing short (11%) and long (7%) periods of activity restriction. This difference did not achieve statistical significance or translate into an appreciable difference in clinical outcome. However, the authors noted the study was underpowered to detect a significant difference in disc reherniation rate and calculated approximately 800 patients per arm would be required to achieve sufficient statistical power.

All previous studies on post-operative restrictions following lumbar discectomy have relied on self-reported adherence to mobility restrictions. Non-adherence is a well-recognized phenomena in spine surgery trials with non-adherence rates in SPORT approximately 40% at one year [8]. Such outcomes are likely to be biased. Contemporary wearable electronic devices that can accurately record the patient’s position (i.e. sitting/standing) enable empirical observation of patient adherence to a regimen of sitting restrictions with great reliability. This trial will be the first to track post-operative adherence to activity restrictions following lumbar discectomy and the impact of adherence on outcomes.

## Methods

### Question

Is the outcome of patients without restrictions inferior to those observing sitting and activity restrictions following lumbar discectomy?

#### Objectives

The study aims to determine whether the outcome of patients without imposed sitting and other behavioral restrictions post lumbar microdiscectomy are inferior to those of patients with imposed restrictions, in terms of disc reherniation, pain and disability outcome measures.

#### Design

**Fig. 1:**
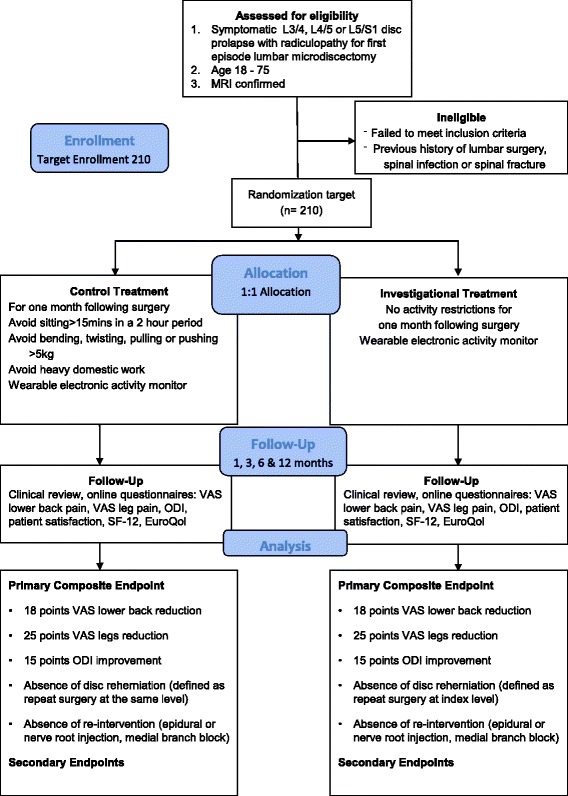
Lumbar microdiscectomy and post-operative activity restrictions trial flow diagram

The will be a randomised controlled surgeon and assessor-blinded trial. The trial design is illustrated in Fig. 1.

### Hypothesis to be tested

That movement restriction following first episode lumbar microdiscectomy in adults results in improved outcomes in disc reherniation rates, pain and disability outcome measures.

#### Participants

The study will consist of patients aged 18–75 years old who meet the inclusion criteria and are undergoing first-episode lumbar microdiscectomy for symptomatic lumbar disc prolapse in the participating private and public hospitals in Melbourne, Victoria, Australia during the trial period. Informed consent will be sought.

Inclusion Criteria.

#### Participants will be

1. Age 18–75 years
2. Suffering from radiculopathy or radicular pain with concordant MRI evidence of lumbar disc herniation at L3/4, L4/5 or L5/S1

#### Exclusion criteria

1. Previous history of lumbar surgery, spinal infection or spinal fracture.

#### Trial sites

The trial is a multi-centre trial conducted in three hospital in Melbourne, Victoria, Australia. The hospitals are as follows:

1. Monash Medical Centre, Clayton, Melbourne, Victoria, Australia
2. Jessie McPherson Private Hospital, Clayton, Melbourne, Victoria
3. Cabrini Hospital Malvern, Malvern, Melbourne, Victoria, Australia

##### Treatment allocation

Patients will be randomised to one of two parallel treatment arms allocated in a 1:1 ratio. Sealed numbered envelopes containing electronically randomised group allocations and group specific post-operative activity instructions will be prepared prior to trial commencement. Following informed consent, a sealed pre-randomised envelope will be allocated by the study nurse to the patient and the patient label affixed to the envelope. The envelope will then be handed to the treating physiotherapist to be opened postoperatively. The study interventions are specifically detailed on this instruction sheet and will be read by the physiotherapist to the patient postoperatively. For both allocation groups, the study card instructions are discussed and reinforced by the treating physiotherapist. The physiotherapist also gives the patient the activity monitor and instructs them on how to use this. The patient will receive a copy of the instruction card to take home and is advised not to disclose their allocation group to medical staff or assessors. The physiotherapist is not involved in subsequent assessment of the patient.

Post-operatively both groups will be fitted with electronic monitoring devices, worn on either the thigh under clothing or carried in the pocket, that will record patient position (sitting/lying/standing) and activity (walking/running/cycling). The devices will be taken off when showering or bathing.

#### Control Treatment

Post-operative activity restrictions represent the traditional standard of care following lumbar microdiscectomy. As such the control group will be advised to follow post-operative activity restrictions for a period of one month following lumbar microdiscectomy. The control group will receive the following specific advice:

For the first one month following surgery:

1. Avoid sitting for longer than 15–30 min in any two hour period
2. No bending, lifting, twisting, pulling or pushing greater than 5 kg
3. Avoid heavy domestic work such as vacuuming, laundry and making beds

And for the first two weeks following surgery:

4. Avoid strenuous sexual activity

The restrictions detailed above reflect post-operative algorithms in current clinical practice. [unpublished data, Daly et al.].

#### Investigational treatment

The investigational treatment arm will be the group without sitting or other restrictions. They will be advised to return to normal activities with no restrictions placed on sitting, exercise, return to work, or other activities as soon as they feel ready.

### Outcomes

#### Primary endpoints

While there is no widely used definition of clinical success following lumbar microdiscectomy it is generally accepted that such a definition should take account of outcome measures such as physical function, disability and pain [9–11]. Intervertebral disc reherniation and reoperation are important considerations as potential primary endpoints. In SPORT[12] 20% of patients who underwent surgery rated their progress as less than a major improvement at one year yet only 6% had undergone reoperation. Thus, reherniation and reoperation alone do not account for the majority of unsatisfactory patient outcomes following lumbar discectomy. The use of a composite endpoint allows for the capture of multiple outcomes that influence the overall success of a clinical intervention while also allowing for increased statistical efficiency and efficient resource utilisation [13, 14].

As such the primary endpoint to be assessed in this trial consists of a composite of the following widely accepted outcome measures:

1. 18 point reduction in VAS lower back
2. 25 point reduction in VAS legs
3. 15 point improvement in ODI score
4. Absence of disc reherniation (defined as repeat surgery at the same level)
5. No other secondary intervention (epidural or nerve root injection, medial branch block)

Using the above definitions of treatment success we would anticipate treatment success rates of ~70–90% in keeping with those reported in the literature [8, 15]. This allows the ability to detect a clinically significant difference in outcomes between the two groups (i.e. rates of clinical success) with smaller groups than required for the detection of differences in recurrence rates (i.e. event rate of “clinical success” of approximately 70–90% as opposed to herniation event rate of 5–10%).

Utilisation of the personal wearable electronic device will enable accurate assessment of patient adherence to the allocated post-operative care group.

#### Secondary endpoints

Secondary endpoints will consist of the following surgical and functional endpoints.

Surgical endpoints:

1. Incidence to 12 months post-operatively of disc reherniation requiring repeat surgery at the same level
2. Incidence to 12 months post-operatively of other parenteral pain management intervention such as epidural or nerve root injection, medial branch block for the primary illness, but excluding enteral or dermal analgesia (fentanyl patches, TENS machine or acupuncture).

Functional endpoints:

1. VAS lower back change score
2. VAS legs change score
3. ODI change score
4. Days to return to work (including days to return to modified duties and to normal duties.)

For functional endpoints, magnitude of change from baseline, adjusted for baseline score will be compared between treatment groups. Additionally, the proportion achieving predefined success thresholds (change scores of 18 for VAS back, 25 for VAS legs and 15 for ODI) will be compared.

### Duration of treatment

Patients will be instructed to follow the post-operative advice- i.e. restrictions or no restrictions for a period of one month. Monitoring of adherence using wearable electronic activity monitors (Activ8, 2 M Engineering, Netherlands) will be for 1 month.

### Follow up schedule

With the assistance of a blinded investigator, participants will complete online outcome questionnaires preoperatively, the day following surgery, and at home at one, three, six and 12 postoperative months.

Questionnaires will include:

1. VAS lower back pain
2. VAS leg pain
3. ODI
4. Current situation/patient satisfaction questionnaire
5. SF-12/EuroQol (EQ-5D)(quality of life questionnaire)

Patients will receive standard post-operative trial-blinded neurosurgical outpatient review at approximately 30–60 days following surgery at trial sites. Teleconsultation will occur at the three, six and 12 months. Patients will only receive further neurosurgical outpatient review if clinically indicated.

### Participant timeline

The participant timeline is illustrated below in Table 1.

**Table 1:**
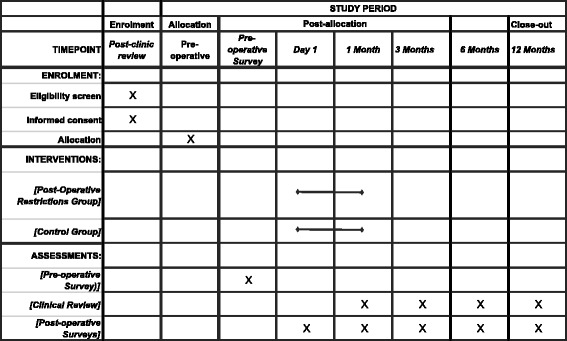
Participant timeline template for schedule of enrolment, interventions and assessment

#### Randomisation and allocation concealment

Randomisation of treatment protocol to sequentially numbered envelopes was performed by an electronic randomisation tool. Sealed envelopes will be sequentially assigned immediately following consent. The treating physiotherapist is given a sealed envelope by the blinded study nurse upon randomisation. This contains the patients assigned group and the appropriate post-operative instructions. This is opened by the physiotherapist post operatively.

#### Blinding

Blinding will be universal from consent till surgery. Postoperatively the physiotherapist will open the envelope and inform the patient of their assigned group. The study nurse will complete all assessment of patients at each time point and will be blinded as to patient randomisation throughout the study. Participants will be instructed not to inform study staff regarding their allocation. Surgical staff will remain blinded throughout the trial.

#### Non-Adherence

Patient data will be analysed on an intention to treat basis. Non-adherence to post-operative activity protocol may be determined by analysis of recorded activity from the wearable device. This will be especially important in light of the trial explaining to participants as part of consent procedures the clinical uncertainty regarding movement restriction, which may result in poor adherence in the group subsequently assigned to restriction.

#### Sample size

As detailed earlier one randomised controlled trial investigating the role of post-operative activity restrictions in outcome following microdiscectomy has recently been reported [7]. The authors recruited 108 patients and noted disc reherniation rates of 11% in the 2-week restriction group and 9% in the 6-week restriction group. Previous studies have indicated a reherniation rate ranging from 2 to 18% [16]. Annual reported reherniation rates have been closer to 4–5% in large series [8, 17, 18]. The authors of the randomised controlled trial calculated it would be necessary to have 800 patients in each arm in order to detect a statistically significant difference in disc reherniation rate and that this may not be feasible. We are in agreement with this assessment.

Reported rates of clinical success for lumbar discectomy vary widely dependent on the criteria. Using the criteria detailed in our composite primary outcome we would anticipate a clinical success rate within the broad range reported in the literature of approximately 70–90%.

In determining the power of this study, we assume that approximately 80% of patients will meet the definition of treatment success. The calculation of sample size can be based upon a threshold of a 20% difference in treatment success as clinically significant (i.e. 80% success vs. 64% success). In order to have 80% power to detect a 20% difference in the binomial outcome of treatment success defined at *p* = 0.05 the sample size calculated would be 78 patients per group. If we allow for an approximately 30% drop-out rate, this will bring the calculated sample size to 105 patients per group for a total of 210 patients in the trial.

### Analyses

#### Baseline characteristics

The baseline characteristics of patients and operative details will be recorded.

#### Statistical analysis

Hazard of reherniation and of parenteral analgesia will be compared by treatment arms. Efficacy will be defined as 1 minus hazard ratio of active vs restricted arms. Efficacy less than absolute delta (see sample size) will be deemed equivalent. Change from baseline adjusted for baseline in functional scores will be compared across treatment arms. Magnitude difference will be compared using ranksum (Mann-Whitney-U) test.

Analysis will report both intention to treat and per protocol results. Per protocol adherence to treatment will compare time in movement by treatment arm using t-test allowing for differential variance**.** Time in movement should differ between arms. Two thresholds will be defined a priori – a sedentary level below which will be considered adherent to movement restriction, and an activity level above which non-restriction will be deemed to have occurred. These thresholds will be used to define adherence. In sensitivity analysis we will examine impact on trial outcomes of excluding those subjects allocated to restriction who moved above this threshold and those unrestricted who were sedentary. We will also conduct sensitivity analysis by cross allocating such subjects.

## Discussion

The longstanding practice of applying post-operative activity restrictions following lumbar spine surgery was based upon the hypothesis that such restrictions may reduce the risk of progressive instability or lumbar disc reherniation [5]. Furthermore, prolonged sitting has been suggested to decrease lumbar lordosis, increase spinal loading and muscle activity and contribute to accelerated disc degeneration and low back pain independent of previous operative intervention [19]. The randomised controlled trial of Bono et al. [7] demonstrated no significant difference in outcome measures between patients who observed two weeks or six weeks of activity restrictions though was underpowered to detect differences in reherniation rates. The only prior studies investigating the impact of removing post-operative restrictions reported no increased risk of reherniation or reoperation in patients not observing activity restrictions following lumbar discectomy surgery but these studies lacked a comparator group and their design was subject to potential bias [5, 6]. Modern lumbar discectomy is now minimally invasive and results in less tissue destruction, further undermining the hypothetical rationale for activity restriction. In a recent survey of Australasian Neurosurgeons, many advised either no sitting restrictions (22%) or sitting as comfort allows (40%) [unpublished data, Daly et al.]. However, the vast majority (84%) advised restricted lifting.

In an uncontrolled prospective cohort study [6] patients who did not observe post-operative activity restrictions returned to work earlier. Mean time to return to work in the cohort was 1.2 weeks. Currently extant recommendations suggest four to 16 weeks off work following lumbar discectomy surgery.

### Activity monitoring/adherence

The activity monitor will monitor and record patient posture (i.e. lying, sitting or standing) and activity (walking, running or cycling) over the one month period following lumbar microdiscectomy. This device is sensitive to acceleration. The validation report described 90.8% correlation between activity monitor output and video analysis [20]. Data is recorded over the one month period on the stand-alone device and then transferred to the study computer.

## Conclusion

Back pain is the leading cause of disability worldwide [21] and intervertebral disc degeneration is a significant contributor to back pain. Lumbar microdiscectomy, performed for symptomatic intervertebral disc herniation, is the most commonly performed spine surgical procedure. Activity restrictions have traditionally been recommended following this operation and patients often advised to delay return to work for four or more weeks with restrictions of similar duration applied to other activities of daily life [6]. Clarification of the role of post-operative restrictions will allow standardisation of post-operative care and potentially allow patients to return to work more rapidly thus reducing the social and economic burden of this condition.
